# Playing with method: testing one approach towards identifying the places of past children

**DOI:** 10.1017/ehs.2020.63

**Published:** 2020-12-14

**Authors:** Mackenzie Cory

**Affiliations:** Department of Anthropology, Indiana University, Bloomington, United States of America

**Keywords:** Childhood, stone circle, Northwest Plains, North America

## Abstract

Before approaching larger questions surrounding the role of children as agents of innovation in the past, we must first be able to confidently archaeologically identify their presence within social spaces. While previous research has broken down some assumptions surrounding the use of material culture by children, there still exists a considerable gap in the identification of features relating to children's activities in the archaeological record. The identification of play areas at archaeological sites contributes to a better understanding of the artefacts located in proximity to them, increasing the accuracy of interpretations of the past. This paper presents a possible methodological solution to identifying children's spaces in the archaeological record of North America's Northwest Plains. The historic record indicates that Indigenous children engaged in domestic play using several varieties of play tipis, some of which have potential to be identified in the archaeological record as small stone circles. I examine if there is any significant difference between stone circles possibly representing play areas from those representing hearths based on the feature attributes identified from archival collections. Analysis of these features from nine Wyoming counties reveals significant differences between hearths and play tipis.

## Graphical abstract

**Figure:**
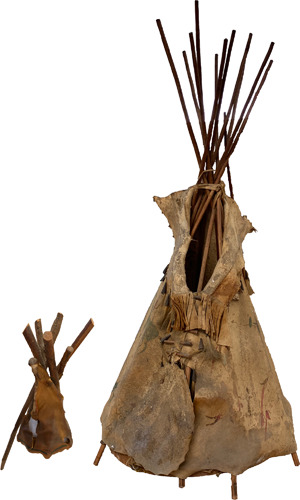

**Media summary:** Ethnographic evidence is used to develop a regional method of identifying children's areas in the NW Plains of the USA.

## Introduction

Children undoubtedly influenced human behaviour in the past, either through their participation in activities within the broader group or through their play with those close to their own age. Despite this fact, it is not common practice to test for evidence for children's activities in the archaeological record, thus removing them from consideration as agents of change. This lack of consideration is unsurprising given the difficulties of identifying items used by children and, perhaps more so, the spaces used by children. Children's activity areas situated close to those used by adults can be easily obscured, or misinterpreted (Langley & Litster, 2018), while those further away from the domestic core may be more ephemeral in nature and fade away without leaving an identifiable trace in the archaeological record, especially in the case of mobile populations. In cases where children and adults are participating in the same activities simultaneously, the interpretive bias towards adults can leave children as non-agentive participants or remove them from the scene altogether unless there is definitive evidence for their presence.

In this paper, I address this issue by testing a new methodology for identifying children's spaces within the context of the Northwest Plains of North America. By examining intrasite feature distribution of small stone circles located in the eastern half of Wyoming – which have the potential to be the remnants of ethnographically recorded miniature tipis used for play by children or left by other adult behaviours (mostly hearths) – this study moves towards building more robust methods of exploring children's spaces in the archaeological record. Ultimately, it appears that size is the primary indicator of differentiated use as variables regarding relation to domestic tipis and the number of stones display no significant difference between features known to be hearths and other small stone circles.

### Childhood research

Historically, archaeologists have devoted few resources to understanding the social roles and activities of children. Lillehammer (1989) first explicitly identified this absence and her ideas were further expanded upon by Sofaer Derevenski (1994a, b, 2000) and Kamp (2001, 2015). Baxter (2005) suggests that this lack of attention is due largely to the mistaken assumption that children are the users of material culture, but not the creators of it. Lucy (2005) attributes part of the problem to the unwillingness of many archaeologists to include children in their interpretations unless there is direct evidence, usually mortuary, of a child's presence.

As it stands, much of the work explicitly addressing children in the past focuses on sedentary populations such as those in Mesoamerica (Ardren & Hutson, 2006; Joyce, 2000), Egypt (Meskell, 1999) or the Iroquois Confederacy (Creese, 2012). In all of these cases the large volume of data available to the researchers allowed for an analysis of childhood as part of a larger population. Sharpe and van Gelder's (2015) examination of finger flutes at Rouffignac is one of the few examples of an investigation of the childhood mobile groups of prehistoric hunter–gatherers. They still, however, use biometric data as a proxy for the physical body of a child to conclude that children were present and active in the space. Langley and Litster (2018) also prove an exception to the sedentary focus in their global analysis into children's material culture, which includes Native North American play tipis, and how children's material culture could be misinterpreted as being ceremonial in nature.

From a more behavioural ecology standpoint, research into children's foraging behaviours display, like the foraging strategies of broader populations, considerable variation greatly depending on the environment in which they live but consistently are either limited or enabled by children's small stature. The dangers of the terrain where foraging takes place can prohibit them from travelling as far as older members of the group. Children still contribute to the resources of their group as they will often compensate for their inefficiency by targeting a wide breadth of more common yet lower-return resources, at times to great success (Kelly, 2013: 190–191).

Regional research into the archaeology of childhood follows a similar pattern. Researchers in the plains and surrounding regions have engaged only occasionally with anthropological childhood research over the last 30 years, generally prioritising sites with mortuary evidence for children such as the Clovis burial cache at the Anzick Site in Montana (Owsley & Hunt, 2001). There are, however, some non-mortuary examples. Dawe (1997) suggests that small, poorly produced projectile points found at the Head-Smashed-In buffalo jump in Alberta may be indicative of toy arrows created by children as these projectile points would not have been effective for hunting owing to their size. Unfortunately, this line of research did not continue beyond the publication of a single article. More recent work dismisses the *a priori* assumptions that result in the exclusion of children from the demographic analysis of domestic and rock art sites in Wyoming (Mackie, 2015) and further west into the Great Basin (Cannon & Woody, 2007) by demonstrating how children impacted the archaeological record through their actions. To the east, Pauketat and Alt's (2005) research at Cahokia has particular relevance for the development of methodological approaches as they work with less certain physical evidence for children occupying spaces in the past and instead rely on postmoulds to suggest that children played a role in the construction of Cahokian architecture based on the depth of the features. Spector's *What this awl means* (1993) not only examined the potential of a worked awl being directly assigned to a girl on the brink of womanhood but also indirectly assigned agency to the broader community of older women who use the awl as a literal and symbolic means of transmitting knowledge.

Regional ethnographies show a similar lack of attention to children. Early ethnographers have long been noted for prioritising the narratives of Indigenous men over the narratives of others. A. B. Kehoe (2012) attributes this androcentrism to these researchers primarily talking to Native men as these would be the only individuals who researchers could easily access for interviews. The first scholarly volume to go beyond this focus and discuss women and children in greater detail was Albers and Medicine's *The hidden half* (1983), which explicitly did so in an Indigenous manner. Despite this progressive shift, children's narratives were combined with the narratives of women, resulting in a focus on how women in the group construct social meaning behind childrearing and not on the experience of childhood itself. Compounding the problem of ethnographically overlooked children in the Northwest Plains is the evidence suggesting that some Northwest Plains groups considered girls to become adults at the onset of puberty, marked by first menses, meaning that girls could be considered young adults in their early teens (Grinnell, 1923). The event marking adulthood was more nebulous for boys as there is no equivalent physical change. Black Elk (Oglala Lakota) suggests that boys could become men after doing something that only a man could do, such as killing a buffalo while in a hunting party or counting coup while accompanying a war party (Neihardt, 1998), a theme reinforced by classic ethnographies detailing other Northwest Plains groups (Linderman, 2002; Lowie, 1935). These cultural requirements for entry into adulthood do not fall into the Western conceptions of adolescence often used as a guide in anthropological research (Schlegel & Barry, 1991).

Two notable ethnographic examples prove exceptions to women and children being overlooked by early researchers. Linderman's (2003) 1930s interviews with Pretty Shield (Apsáalooke) include details of Indigenous girlhood unseen in other works. These details were probably due to Pretty Shield's advanced age and her social status as a healer. MacGregor's (1946) representation of early twentieth-century Lakota childhood in *Warriors without weapons* includes the perspectives of adults reflecting on what constitutes an idealised childhood and the culturally imagined childhood that children would be encouraged to emulate. However, MacGregor also interviews children to determine the lived experience of being a child on Pine Ridge and in the boarding school system.

Broadly speaking, Hirschfeld (2002) views the general absence of research on the history and ethnography of children as a consequence of two processes: an overestimation of the power of adults in enculturation and a lack of understanding of children's culture as a whole, a perspective that resonates with Lancy's (2008) more interdisciplinary work on the nature of childhood. Bluebond-Langer and Korbin (2007) suggest that another possible reason for the lack of research is the challenge of separating research from policymaking in childhood studies, as well as a desire not to rock interdisciplinary boats when drawing comparisons between Western psychological notions of childhood development and ethnographic descriptions. As such, even though there is a general awareness of the locus surrounding childhood research in archaeological and anthropological research, there is disagreement regarding exactly how to address the issue and what data are valid for filling in this gap in understanding

### Stone circles, tipis, and play tipis

Indigenous groups throughout the Northwest Plains relied primarily on the tipi, an all-season conical shelter, for shelter through the Reservation Era with some families continuing to use them today on a temporary basis. These tents used either three or four timber base poles, depending on the group, with another 10–12 support poles that then had a hide or canvas cover wrapped around the frame and anchored with either rocks or stakes (Neuman, 2010). The conical shape of tipis allows them to withstand high winds and heavy snowfall while maintaining a thermodynamic equilibrium in both extreme heat and extreme cold through adjustments of a top flap. During camp moves the poles double as horse or dog travois components that can carry the outer covering as well as the belongings of the tipi's occupants (Laubin & Laubin, 1957). Although early evidence for similar structures occurs at the Frederick level of the Hell Gap site (~8300 BP), tipi-related stone circles become regionally prevalent in the Middle Plains Archaic (4500 BP) and widespread by the Late Plains Archaic (3000 BP; Frison, 1991; Larson et al., 2009; Lugo-Mendez, 2019). Other forms of domestic architecture such as the fully timbered conical lodge, or wikiup, or the semi-subterranean pithouse were used in the survey region but were far less common (Buenger & Goodrick, 2019). The range of conical lodges was largely restricted to the uplands of the Black Hills and Bighorn Mountains, where the vegetation needed to build the frame is more common (C. M. Davis, 2015). Pithouses generally occurred in the intermountain basins towards the central and southwestern portions of the state and are more commonly associated with cultural groups coming from the Great Basin (Smith, 2016).

Early stone circle research was largely limited to understanding the purpose of stone circles in the past (T. F. Kehoe, 1958; Malouf, 1961) before moving towards their utility in anchoring tipis (W. E. Davis, 1983; Tratebas, 1983) and then their connection to camps as domestic spaces (Quigg, 1983; Reher, 1983). More recent research often continues with spatial approaches but with a broader emphasis on understanding the social role of tipis. Scheiber and Finley (2010) approach the issue from the perspective of the domestic household within the larger landscape while Oetelaar (2000) and M. C. Wilson (1995) examine the microcosm of the tipi interior.

The tipi itself formed the core of domestic life in the Northwest Plains with other aspects of domestic life oriented either to the architecture of the tipi itself or to the layout of a small cluster of lodges. Placement of items within the tipi mirrored ontological ideals, as did the placement of the tipi within the camp. The shared communal space surrounding exterior hearths was usually several metres directly in front of the tipi's opening and central to a family group's cluster within the larger camp. Other items such as drying racks or travois could be found to the sides of the tipi, in proximity to them but out of the way of day-to-day activities. Even the process of creating and maintaining tipis required the creation and maintenance of social bonds between families as the task was impossible for any single family.

**Figure 1.**
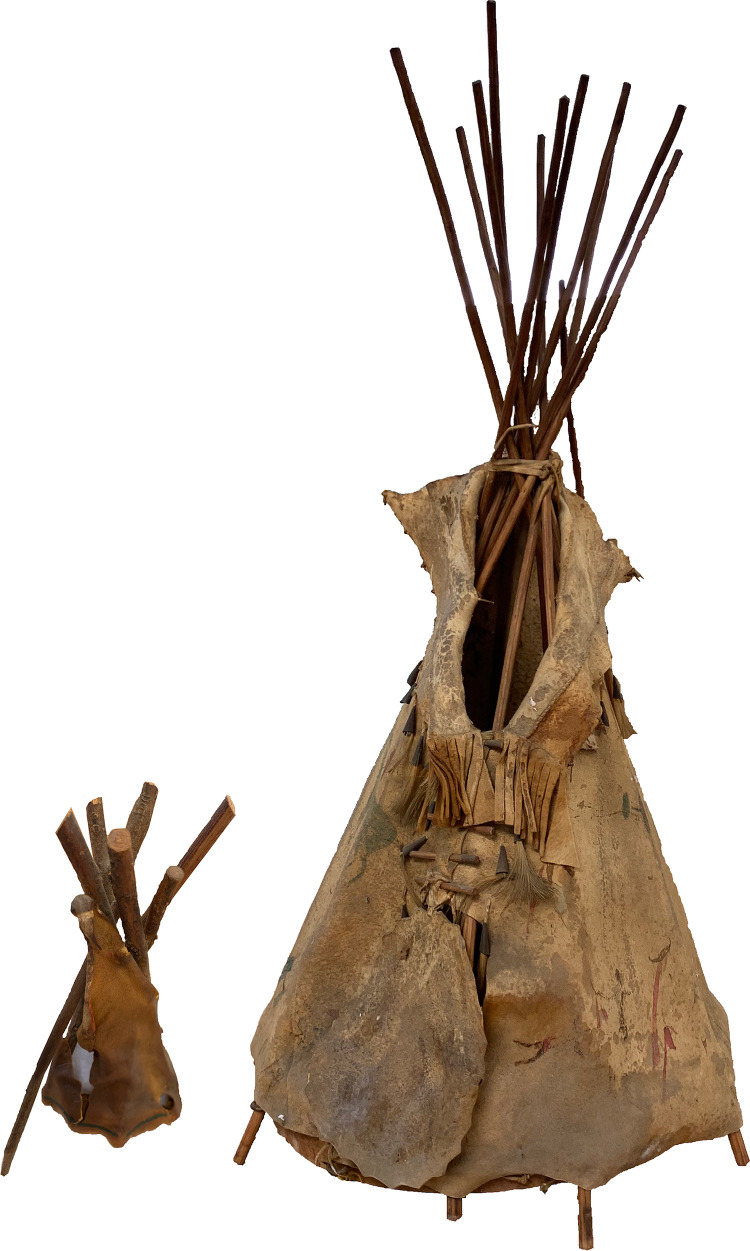
Two varieties of play tipis from the Alice Sheldon's Washakie artefact collection, 1890–1900 (collection number 09686), American Heritage Center, University of Wyoming. Both are Shoshone in origin. On the left is the smallest variety of toy tipi standing only 10 cm tall (box 2, item 3). On the right is an example of the mid-size model tipi standing approximately 60 cm tall (box 4, item 1).

Despite the importance of tipis to our literal understanding of the archaeological and historic records of the Northwest Plains and to the local Indigenous groups who used them, the smaller version used by children is often overlooked. Three varieties of play tipis exist based on evidence from oral histories and historical photographs as well as archived examples. The smallest version measures approximately 10–20 centimetres from the base to the meeting point of the supports with a similar diameter. A mid-size version stands approximately 50 centimetres tall at the meeting point with a base diameter ranging from 30 to 50 centimetres (Figure 1). The largest variety could stand as tall as 2 metres with enough room inside for multiple children. Although the construction of the tipi itself could be the result of either child or adult agency, the repeated use of the object and any subsequent impact on the landscape was almost solely due to children unless the item was repurposed for another task such as a temporary dog house for a litter of puppies or taken apart to patch a domestic tipi.

Although all three types of tipis were generally used for imaginative play, the exact nature of the play varied between the forms. The largest play tipis could be used in a manner akin to a contemporary playhouse in that the child was able go inside the structure. Frequently, several children would use a single play tipi of this sort. The smaller varieties were more similar to dollhouses and could be used in conjunction with other toys by either a single child or a small group. The mid-sized tipi shown in Figure 1 has two dolls, one male and one female, affiliated with it.

The consensus among researchers who have studied the largest variety of play tipi is that although they are significantly smaller than domestic tipis, they are anchored identically (Banks & Snortland, 1995; Grabill 1890; Hail, 1980; T. F. Kehoe, 1958: plate 3, 1960; Laubin & Laubin, 1957; G. L. Wilson, 1921). In this regard, play tipis from before European contact probably would have been anchored with locally sourced rocks while post-contact play tipis would have used either rocks or wooden stakes (Curtis, 1926) to achieve the same result.

Three assumptions guide the analysis presented herein: first, children used play tipis in the past; second, there is a pattern to the play associated with the play tipis; and third, use of play tipis, particularly those using rocks to weigh down the edges, would leave traces visible in the archaeological record. The ethnographic record informs all three assumptions. There is ethnographic evidence for the use of play tipis in traditional play, although no ethnography goes into particular detail about the rules governing children's play tipi usage. Black Elk recounts the shift in his childhood away from playing in the largest size of play tipi with female relatives towards playing boys’ games with unrelated boys from the community (Neihardt, 1998). Pretty Shield describes the personal play tipi of her youth and recalls using the tipi to plug a gap in her group's defences during an attack (Linderman, 2003). Plenty Coup's (Apsáalooke) accounts follow a similar pattern to Black Elk's with him being encouraged by older men to stop playing with small to mid-sized model tipis and start playing games with other boys his age (Linderman, 2002). Grey Bull (Apsáalooke) describes the production of play tipis by girls after boys would hunt calves from nearby buffalo herds and give the hides to friends as a means of impressing them (Lowie, 1935). This account is particularly noteworthy as the girls controlled the use of play tipis after their production, just as adult women would control ownership of domestic tipis (Laubin & Laubin, 1957). Although not explicitly a part of the ethnohistoric record, Dakota anthropologist Ella Deloria's (Yankton Dakota) description of pre-contact Dakota life in *Waterlily* includes the titular character spending time playing with miniature tipis, first with her mother, then with dolls and finally with her younger siblings (Deloria, 2009), although there are some critiques questioning the accuracy of Deloria's writing as it relates to representing the past as she was, in part, writing for a European American audience (Gardner, 2003; Prater, 1995). Mary Sully's (Yankton Dakota) illustrations of various forms of traditional children's play include a drawing of three children assembling and playing with a pair of mid-sized miniature tipis (Sully, n.d.; Deloria, 2019). Play with miniature tipis continued into the boarding school era with evidence of Cheyenne and Arapahoe children using them at the Cantonment Boarding School around 1900 (Montgomery & Colwell, 2019: 180–182) and Lakota children using them at a Pine Ridge school in 1891 (Driving Hawk Sneve, 2016). This type of imitative play is seen both in the past and present throughout the world (Haagen, 1994; Langley & Litster, 2018; Mackie et al., 2015) and it would not be surprising if there are many more examples throughout the region that are not recorded or are hidden in archival accounts.

## Methods

**Figure 2.**
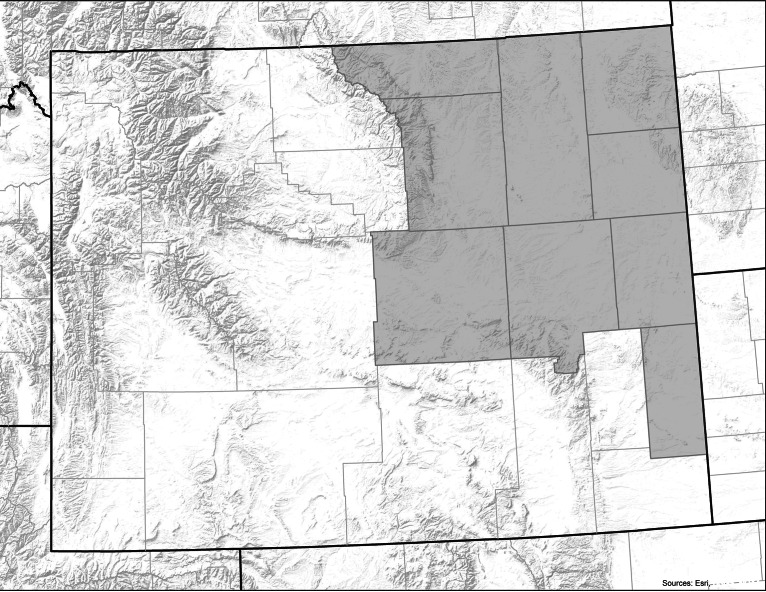
Map of Wyoming with the surveyed counties (Campbell, Converse, Crook, Goshen, Johnson, Natrona, Niobrara, Sheridan and Weston) shaded. (Base maps courtesy of ESRI.)

To investigate if play tipis are visible in the archaeological record, this study utilised a regional dataset as a first test. The survey area includes nine counties in eastern Wyoming with a total area of just over 80,000 km^2^ (Figure 2). Within this area are the traditional homelands of Lakota, Crow and Cheyenne groups as well as neutral hunting grounds used by Blackfoot and Shoshonean groups. The environment is diverse and stone circle sites can be found in shortgrass prairie, riparian zones, badlands, intermontane desert basins, subalpine forests and alpine grasslands. Physiographically the northeastern part of the state is dominated by the Powder River basin with the Black Hills and Bighorn Mountains on the eastern and western peripheries. To the southwest of the survey area is the Wind River basin and the Granite Mountains, giving way to the northern end of the Laramie Mountains and Hartville Uplift in the southeast.

Most of the data was drawn from digital site files stored in the Wyoming State Historic Preservation Office online database. Although these standardised forms have changed over time they generally include legal information necessary for site management, artefact types and counts, feature types and counts and basic descriptions of any archaeological work, as well as photographs and sitemaps. Frequently the file for a single site will consist of multiple site forms reflecting visits by different individuals or companies. These files do not contain the details of mitigation activities which are submitted to the state as site reports and are often unavailable without visiting the state office as they take considerably more time to move from physical to digital formats. These reports contain significantly more detail on any mitigation activities, including better feature maps, and provide a more in-depth examination of the context of the site within the region. The more specific site reports were only used for the evaluation of three sites, the Piney Creek Sites (48JO311 and 48JO312), which are notable for their detailed reporting of small stone circles in relation to domestic spaces (Frison, 1965), and 48GO556 for which I personally recorded feature maps.

Not all of the sites in the survey area were selected for inclusion in the study, only those with previously reported stone circles. Stone circles larger than 2.5 metres were excluded from the sample as they are well within the commonly accepted range of stone circles relating to domestic functions. T. F. Kehoe (1960) argues that the cutoff inner diameter for a domestic stone circle should be 2.1 metres, while Frison (1991) places this limit at 3 metres. Given Finnigan's (1983) research into the impact of taphonomic processes on stone circle creation, specifically the initial outward movement of rocks as they were removed from the tipi's base during the structure's dismantling, I suggest that Kehoe's estimate would result in an internal diameter of less than 1.8 metres, too small for use as a domestic structure, while Frison's estimate is too large to include logistical tipis which would have been large enough to shelter at least one man for short periods of time but small enough for easy transport on dog travois. Owing to these facts, I chose a middling cutoff of 2.5 metres, which is small enough to eliminate all domestic tipis and most logistic tipis. The exception to this rule is a sample of larger stone circles used to demonstrate the size difference between the small stone circles at the core of the study and the more common domestic stone circle.

The small stone circles remaining in the sample had the following attributes coded: (1) the angle of the feature from the opening of the nearest domestic stone circle; (2) the distance from the nearest domestic stone circle; (3) the number of rocks in the feature; and (4) whether the feature demonstrated hearth characteristics. These variables measure trends in play tipi placement and construction observed in historic photographs. Angle and distance were determined from several sources including inclusion on the feature map of a domestic stone circle, location on a larger scaled site map, or written descriptions of the relationship between features of different sizes. The numbers of rocks were coded from a combination of written descriptions, feature maps and photographs. Hearth characteristics include evidence for burning such as oxidisation, staining, charcoal and the presence of fire cracked rock. It should be noted that only a random sample of known hearths was included in the analysis owing to the sheer number of occurrences in the survey area. For features missing one or more attributes, only the attributes present were coded and analysed. Analysis involved descriptive statistics of the dataset and an independent variable *t*-test for statistical significance.

This data, by necessity, is coarse grained. In some cases, the measurements of and distances between features are recorded by the original investigators while others required measuring and coding of the site maps in the reports by hand. Multiple Indigenous groups used the study area and large swaths of it are traditionally considered neutral hunting grounds. As such the archaeological sites within the area represent an accurate sample of those that can be found in the wider Northwest Plains region, including those areas that may have been used more exclusively by one group.

## Results

**Figure 3.**
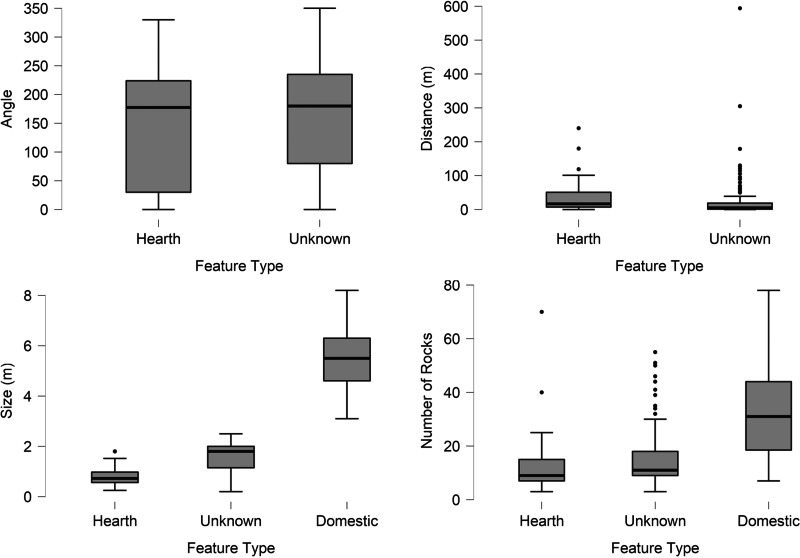
Boxplots of attribute frequencies between hearths, features of unknown origin and domestic features (when applicable). Moving clockwise from the upper left these represent the angle from the opening the nearest domestic stone circle, the distance from the nearest domestic stone circle, the size of the feature and the number of rocks in the feature.

The total number of sites with identified stone circle features in the survey region is 2,077. Of these, 116 sites possessed stone circles under the 2.5 metre internal diameter limit with some sites having multiple features for a total of 226 small stone circles. Table 1 describes the full descriptive statistics of the dataset and these data are illustrated in Figure 3.

**Table 1.** Descriptive statistics of dataset. Hearth features are indicated by H, domestic features by D and those of unknown usage by U

	Angle		Distance (m)		Size (m)			Number of rocks		
	H	U	H	U	H	U	D	H	U	D
Valid	12	35	50	157	50	173	71	31	129	71
Missing	40	138	2	16	2	0	0	21	44	0
Mean	146.58	166.57	35.95	25.19	0.79	1.60	5.54	13.26	15.05	32.25
Standard deviation	115.85	99.39	47.01	63.93	0.37	0.60	1.16	13.12	10.26	15.74
Minimum	0.00	0.00	0.00	0.00	0.25	0.2	3.1	3	3	7
Maximum	330	350	240	594	1.8	2.5	8.2	70	55	78

**Figure 4.**
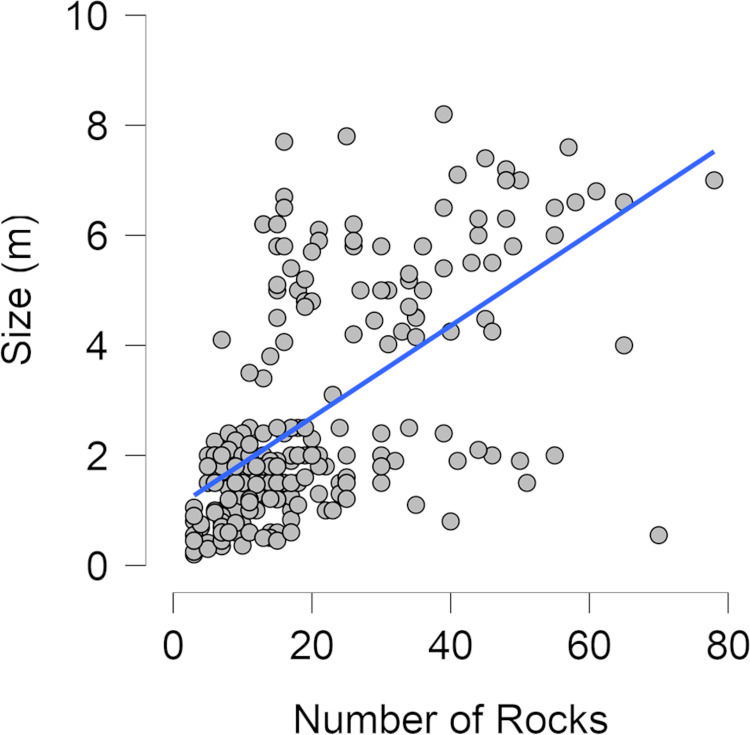
Scatter plot of correlation between the number of rocks and size of the feature in metres. The slope of the regression is 0.08 with an *r*^2^ value of 0.306.

Only 47 features could be correlated to the openings of larger stone circles and there is similar variation between the angles associated with hearth features and features of unclear origin. In both datasets, smaller features were observed at all angles relational to the entrance of the nearest domestic feature with the most common being in front of (0 degrees) the domestic feature's entrance or slightly to the side. Fewer small features were observed to the sides (90 or 270 degrees) of the structures. The distance between the small stone circles and the nearest domestic circle varied greatly for both hearths and unknown features, ranging from an overlap with the outer edge of the domestic circle to several hundred metres away. A majority of both feature types were fewer than 50 metres from the nearest domestic feature with very few surpassing 100 metres from the nearest domestic feature. The size of features produced the clearest difference between the two types. The dataset of features possessing clear hearth characteristics has an average inner diameter 80 centimetres smaller than those without hearth characteristics while the sample of domestic stone circles has a mean of almost 4 metres larger. The data shows little variation between the number of rocks for different feature types. Instead, this attribute appears to be more dependent on the size of the feature and the feature's preservation rather than the feature's original purpose. A test of correlation between size and number of rocks indicates a regression slope of 0.08 and an *r*^2^ value of 0.306 (Figure 4).

The only variable displaying statistically significant differences between potential play areas and hearths was the interior diameter with a Student *t*-test indicating a *p*-value of less than 0.001. As seen in Figure 3, only the largest size of hearth features falls within the middle two quartiles of those features without known function. Likewise, although the lowest size quartile for unknown features overlaps with that of hearth features, a majority of these features are significantly larger, the largest of which possesses a diameter measuring 70 centimetres more than the largest hearth feature. For all other variables the Student *t*-test showed *p*-values ranging between 0.27 and 0.57 with the boxplots also indicating no major differences (Table 2, Figure 3).

**Table 2.** Student's *t*-test of difference between features with hearth properties and other small stone circles

Independent samples *t*-test			
	*t*	d.f.	*p*
Angle	−0.58	45	0.57
Distance (m)	1.10	205	0.27
Size (m)	−9.01	221	<0.001
Number of rocks	−0.83	158	0.41

## Discussion

The analysis of 2,077 sites found that size is the best criteria for determining if a stone circle has potential to have been used as a play area (Figure 3). Three distinct groupings emerge from the data: large stone circles relating to domestic structures, small stone circles with hearth characteristics and a moderate size without hearth characteristics yet too small to be domestic in origin. Features in this final group have the greatest potential to be play areas. Although the size differences between domestic stone circles and smaller stone circles are abundantly clear, the size difference between small stone circles without hearth characteristics and known hearths is helpful in more fully understanding domestic spaces by setting a baseline of what does not constitute a hearth (unless the feature displays clear hearth characteristics). If these features were made by the same patterns of behaviour the expectation is that they would not possess identifiable differences. This is simply not the case. Also important is the evidence that neither the number of rocks in a feature nor its distance from a domestic tipi appear to be helpful in determining the usage of a feature. The number of rocks is far more likely to relate to the size of feature, local availability of lithic resources, and wind strength then to usage while distance to a domestic tipi could be the result of personal preference or taphonomic processes destroying other, closer stone circles.

The method is not without its caveats. One difficulty facing any analysis of stone circle sites is their predominantly atemporal nature. The reoccupation of camps was a regular occurrence and rocks were often removed from older stone circles for use in the construction of new ones, stone circle site formation functioning as a palimpsest with occupations overlapping on the same surface (Morgan et al., 2013). Despite suggestions that the size of stone circles increased through time owing to the introduction of the horse, this is not considered an accurate measure of age as very large stone circles have been dated to the Middle Archaic and small stone circles were created after European contact (M. C. Wilson, 1983). Although there have been successes at using optically stimulated luminescence to date stone circle sites (Feathers et al., 2015), this methodology has not become widespread.

The usefulness of CRM site files cannot be understated as they allowed for the review of over 2,000 sites spread over thousands of square kilometres. However, this source of data is not without its shortcomings. Not all site files are available in standardised formats as these formats did not exist when the Wyoming State Historic Preservation Office first started maintaining their database. For some sites this shortcoming manifested as site files consisting solely of scanned field notes while others simply had a location mapped with no further details. Associated with this issue is the change in reporting standards over time. This change is, of course, a well-known limitation of archival work; however, the approach allows for the inclusion of sites that may have since been destroyed. The sheer number of stone circles across the region combined with their limited potential for understanding chronologies resulted in minimal recording until the late 1970s (L. B. Davis, 1983; Reher, 1983). In these cases, the stone circle was noted, often with an external diameter measurement and an opening if observable, and included in a general site map.

The most apparent limit to analysis is evident in the number of domestic stone circles that do not have a recorded opening angle. Although the relationship between a tipi's opening and another feature is one of the primary factors identified by Banks and Snortland (1995) as useful in differentiating between feature types, there is little physical evidence to support this approach in the archaeological record. This lack of evidence begs the question, what other attributes could be used to differentiate play areas from other similar-sized structures? Sweat lodges would generally be larger and probably further away from the core of the campsite. They may also show evidence of post holes near the edges of the stones and may possibly contain a central depression with fire-cracked rock. Drying racks or stacked travois would leave a much more linear structure than that left by a play tipi, although if leaned up against the side of a domestic structure could leave a feature with a more rounded appearance (Banks & Snortland, 1995). Features coded in site reports as vision quest beds and eagle traps generally have multiple vertical layers of stone to differentiate them from features with a single surface layer. Collapsed cairns may manifest as small stone circles but are more likely to have rocks in the middle of the feature. Hollow cairns tend towards fewer rocks in the interior but also have a notable depression in many instances (Amundsen-Meyer & Leyden, 2020). In addition hollow cairns are far more common in parts of the Northwest Plains located to the north of the survey area. This leaves the difficulty presented by hearths. As noted in the methodology, features possessing oxidisation, staining, charcoal or fire-cracked rock can easily be coded as hearths. However, the non-depositional environment of the Northwest Plains reduces the likelihood of staining and charcoal remaining in association with features.

In addition, the recording requirements for legally defining a site do not necessarily correlate with actual landscape understandings in the past or present (Alberti, 2016). This issue has been previously raised in archaeological research through the idea of ‘total landscape’ (Colwell & Ferguson, 2014) or the use of Traditional Cultural Properties and archaeological districts to document groups of sites (Zedeño et al., 1997). While these ideas are occasionally implemented at larger sites in the surveyed counties such as 48GO398 or 45NA2457, smaller clusters of features are often labelled as separate sites and managed as such. Although these smaller sites are part of larger archaeological landscapes, the proposed methodology does not take the broader landscape into account, resulting in a fragmented understanding of the relationships between distant features and a bias against smaller features that are further away from camp areas. This, in addition to the preservation and identification biases, prevents just under 10% of the potential features from having accurate measures of distance from a domestic feature.

Many of these difficulties can be mitigated through a finer grained analysis of local data. Although the overall number of features would be smaller than a regional dataset, many areas within the survey region possess hundreds of stone circle features. This is still enough for a statistically significant dataset. Local analysis also allows sites to be either re-visited to fill in the gaps present in the state databases or for a more thorough initial recording of sites that avoids some of the pitfalls altogether. In addition, coding the full sample of hearths in the survey area and historic photographs with known locations will significantly increase the number of features with a relationship to the opening of domestic stone circles, improving the accuracy of that variable. This is perhaps the most important change to make as it directly relates to understanding the placement of play areas as it relates to the broader ontological systems of historic Indigenous groups, working towards answering questions of why in addition to where.

## Conclusion

The identification of a significant size difference between hearth features, domestic features and features of unknown origin, although seemingly minor, represents a significant step forwards towards the identification of children's areas in the archaeological record of the Northwest Plains. This study helps to elucidate childhood in the past by demonstrating the utility of using historic photographs to generate informed archaeological tests. Although not all attributes display significant differences, the method shows potential, especially if combined with a finer-grained test for further refinement.

Even with our multidisciplinary understandings of the experiences impacting children throughout time globally, we face regional constraints that necessitate diverse means of identification. There is no universal approach that can identify children in the archaeological record despite childhood being a near universal stage in the development of social personhood. In the case of this project, the nomadism of the region's Indigenous groups combined with the fact that dogs were the largest pack animal until the 1500s limits the amount of material that could be carried by a family and therefore the amount of material found in the archaeological record is also limited. For this reason, I turn to features instead of artefacts.

As discussed elsewhere in this Special Issue, the interdisciplinary field of child studies is shifting towards a more robust interpretation of the role children played in the past, yet this goal is impossible to achieve without first identifying the physical and temporal spaces children inhabited and where their activities occurred. In doing so, we generate positive feedback in which we can more confidently identify artefacts and in turn potentially identify more features based on intrasite spatial relationships. As our theoretical understandings of prehistoric childhood improve so do our methods. Only after we are confident in our understanding of where children were and where they were not can we determine how and where children functioned as actors in cultural and technological innovation, ultimately trying to understand their actions through their unique lenses.

## Data Availability

The data that support these findings are part of a dissertation project and will be available in IUScholarworks at https://scholarworks.iu.edu/ following a maximum of 1 year embargo from the completion of the dissertation research. Site location information will be withheld for legal reasons.
